# Experimental and Numerical Sensitivity Assessment of Viscoelasticity for Polymer Composite Materials

**DOI:** 10.1038/s41598-020-57552-3

**Published:** 2020-01-20

**Authors:** Mohammad Mahdi Javidan, Jinkoo Kim

**Affiliations:** 1 Research assistant, Department of Civil & Architectural Engineering, Sungkyunkwan University, Suwon, Republic of Korea; 2 Professor, Department of Civil & Architectural Engineering, Sungkyunkwan University, Suwon, Republic of Korea

**Keywords:** Engineering, Materials science

## Abstract

Viscoelastic polymer composites are widely used for vibration control in different fields of engineering like aerospace, mechanical, and structural engineering. The viscoelastic properties of these materials are strain rate-dependent and are highly related to frequency. Yet to date, less attention has been paid to quantifying the effects of these parameters and their interactions on damping properties and providing an approximation method for further applications. In the present research, a series of experimental tests was conducted on a viscoelastic material and the experimental data were numerically analyzed in detail. Sensitivity analyses are usually applied to quantify uncertainty using sampling techniques. However, in this study a method was proposed to derive a closed-form solution using the response surface function and a derivative-based global sensitivity analysis to evaluate the output contribution of each parameter. These effects were quantified and several approximation statistics were provided for future engineering implementations. The computational evaluation conducted in this study gives a detailed insight into the mechanical behavior of viscoelastic materials.

## Introduction

Viscoelastic polymer composite materials or, in short here, viscoelastic materials (VEMs) are effectively used to attenuate the dynamic response of various devices and structures and dissipate the acoustic energy due to their viscoelasticity and damping properties^1–7^. Having a quite accurate estimation of these properties and their mechanical behavior is of paramount importance in engineering design. However, the key point about the application of VEMs is their complex behavior due to the strain-rate dependency which can be also unknown prior to the design. Hence, there is a need to quantify these effects and provide a confidence interval for practical implementation of these materials. The main factors affecting the mechanical behavior of VEMs are excitation frequency, strain amplitude, number of cycles, and ambient temperature. The storage modulus of reinforced vulcanized elastomers decreases as a function of strain amplitude, and the loss modulus shows an initial increase but decreases afterwards, which is called the Payne effect^8,9^. Many experimental studies have been carried out to evaluate the effects of these factors^10–14^ and they showed that the damping properties of VEMs are more or less sensitive to these factors. Chang *et al*.^14^ stated that the load frequency and the temperature have significant effects on dynamic characteristics. However, no significant effects were observed for strain amplitudes limited to 0.5 in their designed experiment. Therefore, the effects of strain amplitude was not considered in derivation of empirical functions^14^. Eftekhari and Fatemi^15^ studied the influence of loading frequency on the fatigue behavior of several composites under constant amplitude fatigue test. They developed an analytical model to take into account the effects of frequency, stress, temperature, and fiber orientation on the fatigue life. Lino *et al*.^9^ proposed a phenomenological model which takes into account the Payne effect, and describes the storage and the loss moduli as the function of frequency and amplitude.

Although effects of strain amplitude on VEMs can be considerable, the main approach in the previous research has been conducting parametric studies and comparing the results. As mentioned earlier, less attention has been paid to quantifying these effects to consider uncertainties in design. Providing a reasonable approximation and metamodel for damping parameters under a specific condition is also missing, and therefore design of devices like dampers using VEMs are complicated and mostly iterative^16^. In this study an approximation is made using the response surface metamodel, and a closed-form solution is proposed which quantifies the exact effect of the strain amplitude and frequency on the moduli using sensitivity analysis and the response surface.

There are some studies on the sensitivity of damping properties to the layer thickness, temperature, or fiber orientations using numerical and analytical methods^17–19^. Sensitivity tests are generally utilized to identify the most influential parameters on an output response and the range of output variation due to them^20–24^. These results are used to approximate the variation in the output response, or its uncertainty which can be considered in analysis or design process. On the other hand, they can be used in control of the output response by monitoring the influential factors or in model simplification. These studies showed that the damping properties can be changed due to the layer thickness and the fiber orientations can influence the hysteresis stress-strain curve^17^.

## Scope and Limitations

**Figure 1:**
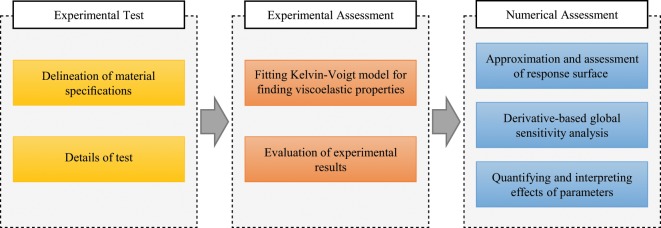
The framework of the present study for evaluating the effects of strain amplitude and frequency on damping properties of VEMs.

In this research, a series of experimental tests was carried out to evaluate the effects of excitation frequency and strain on the damping properties of a viscoelastic polymer composite. As shown in Fig. 1, the framework of the present research can be divided into three sections. In the first section, the experimental test, material specifications, and other details are described. The viscoelastic properties are then determined using the Kelvin-Voigt model and statistical methods in the next section. The experimental data are evaluated based on a parametric study approach and the effects of frequency and strain amplitude on viscoelastic properties are assessed. A more in-depth numerical assessment of these parameters for the next part is carried out by approximating the response surface function, which can also provide a tool for further applications. In addition to ordinary statistical analyses of experimental parameters, the effects of each factor were quantified using the fitted response surface and a derivative-based global sensitivity analysis which is called the Morris method^25^.

Sensitivity analysis is generally performed in the context of probability theory, and is done with sampling techniques. However, sensitivity analysis is applied in this study with some modification, and a method is proposed to study the overall response surface using integration. The sensitivities are derived as a closed-form solution, and the implications are presented and discussed. The results of this sensitivity analysis quantify the exact effects of both frequency and strain amplitude all over the experiment domain, which can be utilized to estimate the damping properties of VEMs in practical implementations.

The main scope of this research is to study and quantify the effects of strain amplitude and frequency on viscoelasticity of polymer composite materials, and provide a sensitivity method for this end. Nevertheless, this research is carried out in a limited range of input parameters and only on one type of carbon-filled elastomer. The strain amplitudes are between 0.1 and 1.2 and the frequency is applied in a range of one decade between 0.05 Hz and 0.5 Hz which is practical frequency range in motion control of building structures. It should be noted that the results and observations are made based on the test domain, and might be changed in different strain and frequency ranges.

In this study the Kelvin-Voigt model is applied to describe the behavior of the vulcanized elastomer due to its practicality and wide application in the structural engineering field. As this model does not take the stress relaxation into consideration, it might be insufficient under the condition of large strain amplitudes. However, Kelvin-Voigt model can be easily utilized in most of the structural analysis softwares without difficulties. The provided supplementary dataset is also of interest for further use.

## Experimental Test

### Material specifications and experimental setup

**Figure 2:**
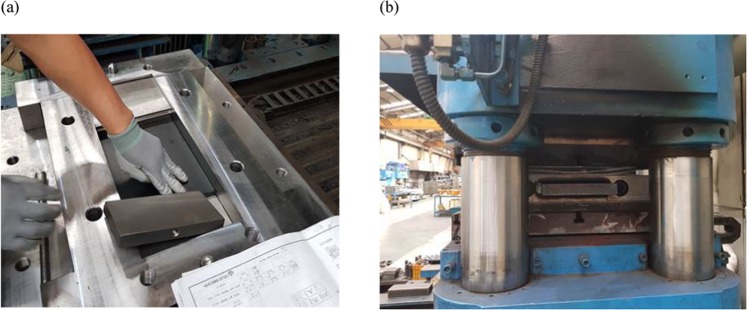
(**a**) Assembly of the VEM pad, steel plates, and the mold. The mold was first preheated at 135 °C for one hour and then the parts were assembled in the mold. (**b**) Molding process of the test specimen. After assembling the material and the steel plates in the preheated mold, the molding process was done at the temperature of 135 °C for two and a half hours. Lastly, the specimens were separated from the mold and cleaned for the test.

The composition specifications of the VEM tested in this research is shown in Table 1. The VEM is generally provided in the form of rectangular pads and is utilized under shear actions. The most common configuration is to attach two VEM pads between three steel plates, two outer plates and one moving plate at the center. Two test specimens were manufactured and the VEM pads were bonded to the steel plates by the molding process at the temperature of 135 °C for two and a half hours as shown in Fig. 2.

**Table 1:** Composition specifications of the VEM used in the experiment.

Component	Ratio	Component	Ratio
Natural rubber	26.0%	Stearic acid	0.5%
Synthetic rubber	13.0%	Antioxidant	3.9%
Liquid rubber	13.0%	Accelerator	0.6%
Carbon black	40.1%	Sulfur	0.3%
ZnO	2.6%

**Figure 3:**
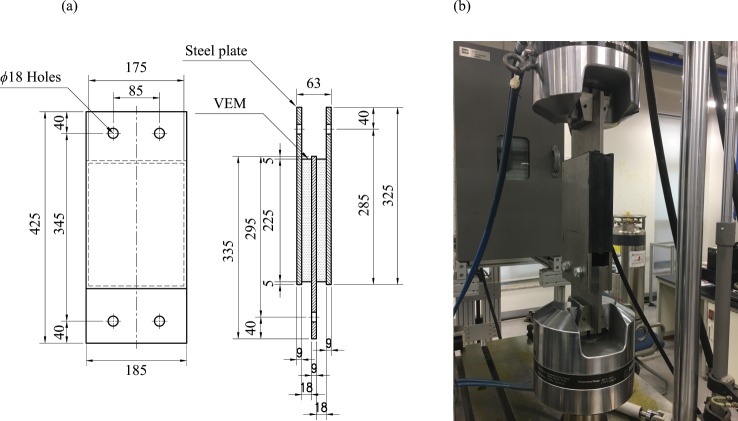
(**a**) Dimensions of the test specimens (unit: mm). (**b**) Test setup. Details of the installation jig with a 500 kN actuator acting on the test specimen.

The dimensions of the test specimens and the test setup are shown in Fig. 3. The two specimens were tested under 3 frequencies, 7 displacement amplitudes, and 12 cycles at the room temperature between 18 °C and 23 °C. The frequencies were *f*_*i*_ = 0.05 Hz Hz, 0.5 Hz, and 0.5 Hz, and the first displacement amplitudes *a*_1_ was 1.8 mm corresponding to the strain amplitude of 0.1 and other amplitudes were determined by,

1 $${a}_{j}=2(j-1){a}_{1},$$

where $2\le j\le 7$. Therefore, 24 combinations of frequency and amplitude were applied to the two specimens under 12 cycles and the data were obtained. The amplitudes and frequencies of the experiment were designed based on most commonly used parameters and operability range of the actuator, including the maximum loading rate and stroke.

## Experimental Results

**Figure 4:**
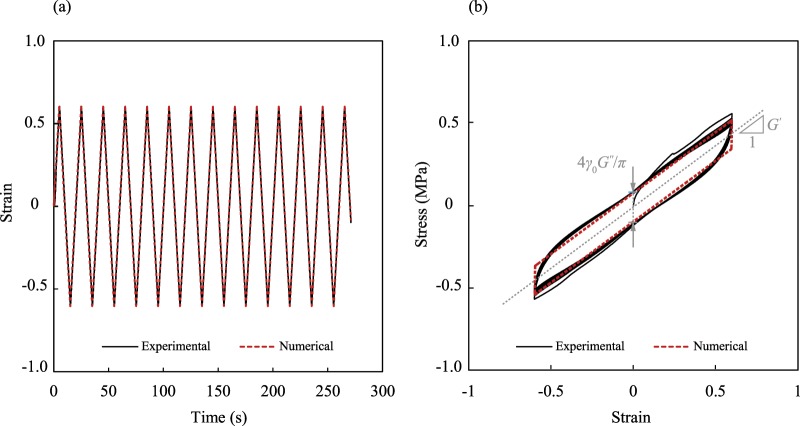
(**a**) Applied strain to the test specimen under the strain amplitude of 0.6 and the frequency of 0.05 Hz and its numerical simulation using the triangle wave function. Despite the fact that the nominal values of frequency and amplitude were known, the two parameters were fitted to get the closest estimation of the applied strain. (**b**) Comparison of the experimental and fitted numerical stress-strain curve of the VEM. The storage modulus defines the slope of the hysteresis curve and the loss modulus specifies the stress intercept while unloading.

The mechanical behavior of VEMs can be represented by a parallel combination of linear elastic and linear viscous behaviors^26,27^, which is called Kelvin-Voigt model. Accordingly, the relation between shear stress 𝜏 and shear strain γ is expressed by,

2 $$\tau =G^{\prime} \gamma +\frac{G^{\prime\prime} }{\omega }\frac{d\gamma }{dt},$$

where *G*′ and *G*′′ are respectively storage and loss moduli with the unit of shear stress (MPa(N/mm2); *ω*(rad/s) is the angular load frequency. The shear stress and strain were calculated using the experimental force-displacement and the dimensions of the VEM pads. Strain is dimensionless and is equal to the ratio of the shear deformation of the VEM pad to its thickness (mm/mm). Compared to an ordinary sinusoidal function and Fourier expansion, the triangle wave was deemed to give better approximations of the imposed strain, which is determined as,

3 $$\gamma ={\gamma }_{0}\frac{2\omega }{\pi }(t-\frac{\pi }{\omega }\lfloor \frac{\omega }{\pi }t+\frac{1}{2}\rfloor ){(-1)}^{\lfloor \frac{\omega }{\pi }t+\frac{1}{2}\rfloor },$$

where *γ*_0_ is strain amplitude and *t* is time. The first step in analyzing the effects of strain amplitude and frequency on the damping properties, is to obtain the storage and loss moduli with a good accuracy. To this end, the experimental strain time history for each of the 42 tests was numerically applied to Eq. 2 and the moduli were determined in such a way that the numerical stresses were fitted to their corresponding experimental values. Each test contains 11264 to 14453 data points for shear stress with a total number of 561073 for the whole 42 tests. For each test, the storage and loss moduli were obtained by minimizing the sum of squared errors of stress prediction using the generalized reduced gradient algorithm^28^. For instance, the applied strain time history and the corresponding stress-strain curve for the strain amplitude of 0.6 and the frequency of 0.05 Hz along with the numerical results are depicted in Fig. 4.

The accuracy of numerical shear stresses obtained by finding the moduli and fitting Eq. 2 to the whole experimental data was quantified in Table 2. The statistics are mean absolute error (MAE), root-mean-square error (RMSE) and the normalized one using interquartile range (RMSEIQR), Pearson correlation coefficient (Pearson’s *r*), and coefficient of determination (*r*^2^). The MAE and RMSE show the average error of 0.019MPa and a standard deviation of 0.032 MPa. This variation in error is in the order of 8.5% based on the RMSEIQR which is analogous to the coefficient of variation. Pearson’s *r* and *r*^2^ are also quite close to 1.0 and 100% and it shows that the numerical model with the obtained moduli fits well to the experimental data. The remaining error is partly due to imposing the predetermined strain function of Eq. 3 to the numerical model, and also partly due to the strains with large amplitudes under the scragging condition. Scragging is a change in the molecular structure of elastomers under large strains so that they show more stable hysteresis behavior at lower strain levels^29^. As mentioned, fitting the numerical model to the experimental data was done in a point-by-point way by minimizing the sum of squared errors of stress prediction. In addition to the point-by-point comparison, it is possible to determine the storage and loss moduli using the area of the stress-strain curves especially under large strains^30^. Even though the Kelvin-Voigt model does not consider the stress relaxation in the carbon-filled elastomers under large strain amplitudes, it is used here due to its simplicity and wide application in engineering. For further research, the Maxwell-Wiechert model or Bouc-Wen model can also be applied. The statistical measures provided in Table 2 shows that the fitted numerical stresses using the obtained moduli are in a complete agreement with the corresponding experimental stresses, and the moduli can be used for further analysis.

**Table 2:** Goodness of fit of shear stress using the numerical model.

Number of data	MAE	RMSE	RMSEIQR	Pearson’s r	*r*^2^
561073	0.019 MPa	0.031 MPa	8.5%	0.994	98.8%

**Figure 5:**
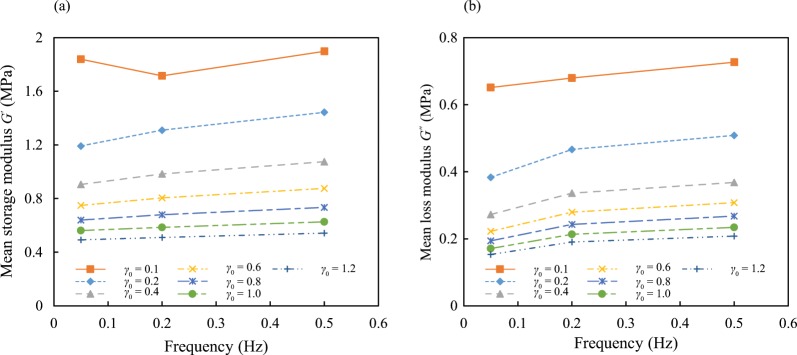
(**a**) Mean storage modulus *G*′ versus load frequency at different strain amplitudes; (**b**) Mean loss modulus *G*′′ versus frequency at different strain amplitudes.

The crude experimental results of the storage and loss moduli are summarized and demonstrated in Fig. 5. The calculated moduli under the same frequency and strain amplitude for the two test specimens were quite close and they were averaged and reported as mean moduli. It is observed at the first sight that the influence of strain amplitude is significant on both storage and loss moduli, and the frequency has a relatively subtle effect on the moduli compared to the strain amplitude. The frequency has a slight influence on the storage modulus and can increase it more clearly at smaller strain amplitudes. In contrast, the strain amplitude *γ*_0_ can significantly affect the storage modulus, and the effect of loading frequency on the loss modulus is more noticeable. Similar to the storage modulus, the strain amplitude considerably affects the loss modulus. Increase in the strain amplitude results in considerable decrease in the storage and loss moduli, which is the well-known Payne effect^8,9,31^. The effects of frequency on the loss modulus is more tangible and it has a slight nonlinearity, but in general the load frequency has a relatively linear effect which is also negligible at higher strain rates. The relation between the strain amplitude and the moduli is completely nonlinear, and changes in strain amplitude at higher values have subtle effects on the moduli. Taking into account these relations is essential to properly generate the response surface function in the next section.

## Numerical Assessment

### Response surface

Different methods and techniques^32^ can be utilized to approximate the response surface function. These methods include response surface methodology^33^, Bayesian networks^34,35^, neural networks^36,37^, and other machine-learning techniques^38,39^ which have been implemented in material science. The main advantage of machine-learning techniques is that many of them are non-parametric and a priori equations are not required to describe the response of interest. However, the response surface methodology provides a great simplicity and it can be easily treated while deriving a closed-form solution. The approximated response surface can be directly studied and it sometimes need a smaller number of samples compared to probabilistic machine-learning methods.

**Figure 6:**
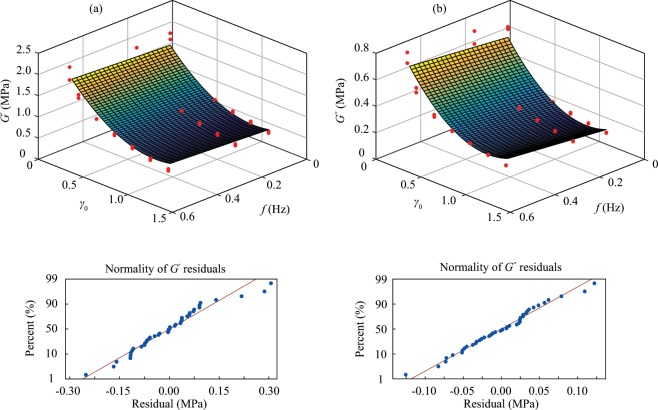
Fitted response surfaces of the moduli, observed data points and the normality test of their residuals: (**a**) Storage modulus *G*′. (**b**) Loss modulus *G*′′. In spite of three outliers for each modulus related to high strain amplitudes, the normality tests of residuals show that there is no heteroscedasticity and the error is consistent for all the observed data. Hence, the fitted response surfaces can approximate the moduli with the same predictability all over the range of the experiment.

In the present research, the response surface methodology is utilized. Full-quadratic functions of two independent variables were considered to approximate the response surface functions of the moduli. It should be mentioned that the response surface methodology is an approximate technique, and thus the use of it does not necessarily imply that the relations between the moduli and input parameters are quadratic or linear against another parameter. It was observed that the second-order terms for frequency and its interaction with strain amplitude are redundant with p-values greater than the significance level *α* of 0.05. Therefore these terms were omitted to reach low-order polynomials to avoid overfitting and this complies with the observed experimental data described in the previous section. As was observed in experimental results, the relation between the moduli and frequency is almost linear while it is nonlinear for the strain amplitude. Accordingly, the response function can be approximated by,

4 $$G^{\prime} \,{\rm{MPa}}=1.8654+0.2657f-2.6630{\gamma }_{0}+1.274{\gamma }_{0}^{2},$$

5 $$G^{\prime\prime} \,{\rm{MPa}}=0.6771+0.1710f-1.1059{\gamma }_{0}+0.5687{\gamma }_{0}^{2},$$

where *f* is load frequency and equal to $\frac{\omega }{2\pi }$. The p-value for all terms are zero which implies that the effects of these terms are significant except the frequency for the storage modulus which is 0.009 and much lower than 0.05. More detailed statistics for checking the accuracy of the models are summarized in Table 3. The standard deviations of the difference between the data points and the fitted values are denoted as *S*, which are adequately small compared to the values of the moduli. The values for coefficient of determination *r*^2^ are greater than 90%, which shows that the fitted response surface functions have enough accuracy. Since there is no much difference between *r*^2^ and the adjusted *r*^2^, it is ensured that the data are not overfitted and the high predicted *r*^2^ prove the capability of predicting for the response surface functions. The approximated response surfaces of the moduli and their observed data points with the normality test of residuals are shown in Fig. 6. It can be seen that the generated functions can adequately approximate the response surfaces.

**Table 3:** Checking accuracy of the response surface functions.

Property	S (MPa)	*r*^2^ (%)	adjusted *r*^2^ (%)	predicted *r*^2^ (%)
Storage modulus	0.117	93.6	93.1	91.9
Loss modulus	0.053	91.2	90.5	89.1

The intercepts of the response surface functions, i.e. *G*′ = 1.86 MPa and *G*′′ = 0.68 mpA, show the amount of damping properties for low frequencies and small strain amplitudes. These values can be slightly increased by the first-order terms of frequency which are called the main effects. The main effects of strain amplitude on the moduli are negative, thus decreasing the damping properties. Due to the positive second-order term of strain amplitude, this effect is nonlinear and by increasing the strain amplitude, its influence is decreased. It is worthwhile to mention that based on the response surface functions, there is no noticeable interaction between the frequency and strain amplitude in the considered experiment domain. As mentioned earlier, the interaction terms of the fitted response surface were omitted due to their high p-values which show their insignificancy and redundancy to the estimated model.

### Sensitivity analysis

The abovementioned effects on the viscoelasticity can be quantified all over the experiment domain by a derivative-based global sensitivity which is called the Morris method^25^. In general, this method is performed using the Monte Carlo simulation; however, a closed form solution is derived in this study using the response surface functions. In this method, random samples are chosen from the input space. For each sample point, one input variable at time is subjected to an infinitesimal perturbation and the first-order derivatives of the function is calculated using the finite-difference method. The function is perturbed with respect to next input variables successively which characterize an infinitesimal trajectory. The first-order derivative for each variable is obtained at the sample point, which is called the elementary effect^40,41^ and the procedure is repeated for all samples. The mean elementary effect *μ* for each variable all over a considered domain is the first parameter of interest. Since the elements can cancel out each other, the mean absolute elementary effect μ^*^ is usually determined. Using a closed-form solution, μ^*^ for the effect of frequency on the storage modulus as an example can be determined by

6 $${\mu }^{\ast }=|\overline{\frac{\partial G^{\prime} }{\partial f}}|=\frac{1}{{\iint }_{U}dfd{\gamma }_{0}}{\iint }_{U}|\frac{\partial G^{\prime} }{\partial f}|dfd{\gamma }_{0},$$

where *U* is the experiment domain. This parameter quantifies the average rate of change in the storage modulus due to the frequency all over the domain of the response surface. Since the response surface functions are numerically approximated and available, the above mentioned definite integral can be easily calculated for both moduli and either frequency or strain amplitude. The standard deviation of elementary effects is the second important parameter, and shows the nonlinearity of changes in the response surface with respect to an input variable. As an example, *σ* for the frequency and the storage modulus is obtained by

7 $$\sigma =\sqrt{E[{(\frac{\partial G^{\prime} }{\partial f}-\mu )}^{2}]}=\sqrt{\frac{1}{{\iint }_{U}dfd{\gamma }_{0}}{\iint }_{U}{(\frac{\partial G^{\prime} }{\partial f}-\mu )}^{2}dfd{\gamma }_{0}},$$

which is equal to zero for frequency here due to the linearity of the response surface function with respect to this parameter. The results of the sensitivity analysis is shown in Table 4.

**Table 4:** Morris method parameters for the damping properties of the VEM.

Property	Frequency		Strain amplitude	
	${{\boldsymbol{\mu }}}^{{\boldsymbol{\ast }}}\,(\frac{{\bf{MPa}}}{{\bf{Hz}}})$	${\boldsymbol{\sigma }}\,(\frac{{\bf{MPa}}}{{\bf{Hz}}})$	${{\boldsymbol{\mu }}}^{{\boldsymbol{\ast }}}\,(\frac{{\bf{MPa}}}{{\bf{1}}})$	${\boldsymbol{\sigma }}\,(\frac{{\bf{MPa}}}{{\bf{1}}})$
Storage modulus	0.266	0	1.041	0.809
Loss modulus	0.171	0	0.420	0.361

The results of the sensitivity analysis show that the rates of the change in the storage and loss moduli to change in the frequency are on average $0.266\,\frac{{\rm{MPa}}}{{\rm{Hz}}}\,\,$and $0.171\,\frac{{\rm{MPa}}}{{\rm{Hz}}}$, respectively. According to the standard deviation, these rates do not change in the range of the experiment characterized by the fitted response surface functions. Compared with the frequency, the effects of the strain amplitude are much more significant. The average rates of change in the storage and loss moduli due to the strain amplitude are respectively 3.91 and 2.46 times the effects due to the frequency. It was observed in the previous sections that the strain amplitude decreases the moduli, and it is found that for a unit increase in the strain amplitude, the storage and loss moduli decrease on average by $1.041\,\frac{{\rm{MPa}}}{1}$ and $0.420\,\frac{{\rm{MPa}}}{1}$, respectively. These rates can vary with the standard deviations of $0.809\,\frac{{\rm{MPa}}}{1}$ for the storage modulus and $0.361\,\frac{{\rm{MPa}}}{1}$ for the loss modulus. Based on the previous assessment, it is clear that the rate of change is high at small strain amplitudes and becomes less effective at higher strain rates. It is worthwhile to mention that the rates of change in the effects of strain amplitude and frequency are determined in this study, and should not be misunderstood with the effects themselves. These statistics can sufficiently and quantitatively describe the effects of frequency and strain amplitude on the viscoelasticity, and provide detailed insights not only into the mechanical behavior of the tested VEM, but also other VEMs with similar specifications.

### Concluding remarks

In the present research, the effects of loading frequency and shear strain amplitude on the damping properties of a viscoelastic polymer composite material were evaluated and quantified using cyclic loading tests and numerical methods. Two specimens were prepared and tested under 3 frequencies and 7 strain amplitudes. The material specifications and instrumentation of the experiment were described in detail and the experimental results for the storage and loss moduli were discussed. The numerical assessment of these effects were carried out using the response surface methodology and a derivative-based global sensitivity analysis called the Morris method. The response surfaces of the storage and loss moduli were approximated using quadratic functions and their accuracy were ensured using statistical measures. These functions were studied and further utilized to derive a closed-form solution for quantitative assessment of frequency and strain amplitude effects.

The results showed that the shear strain amplitude had significant effects on the moduli and mechanical behavior of the considered viscoelastic material. On the other hand, the loading frequency is less effective compared to the strain amplitude. Based on the response surface functions, it was observed that there was no noticeable interaction effects between the frequency and the strain amplitude on the moduli. Based on the results of the sensitivity analysis, the rates of the change in the storage and loss moduli to change in the frequency were on average $0.266\,\frac{{\rm{MPa}}}{{\rm{Hz}}}\,\,$and $0.171\,\frac{{\rm{MPa}}}{{\rm{Hz}}}$, respectively. For a unit change in the strain amplitude, the storage and loss moduli decreased on average by $1.041\,\frac{{\rm{MPa}}}{1}$ and $0.420\,\frac{{\rm{MPa}}}{1}$, respectively. These rates changed over the experiment domain with the standard deviations of $0.809\,\frac{{\rm{MPa}}}{1}$ for the storage modulus and $0.361\,\frac{{\rm{MPa}}}{1}$ for the loss modulus.

It should be noted that the conclusions drawn here are based on the data obtained at the test domain, and might be changed for data obtained out of this range. However, the experimental results, approximations, and quantitative measures provided in this research can be used for practical applications, and the discussed methods can give insight into the further research on viscoelastic polymer composites.

## Methods

Fitting the Kelvin-Voigt model to the experimental data was performed using the generalized reduced gradient algorithm provided by the Solver add-in of Microsoft Excel, and the data were provided as supplementary information files to ensure reproducibility. Conducting the statistical analyses on the data was done using MATLAB and the response surface functions were created and evaluated using the Minitab statistics package.

## Data Availability

The authors declare that the experimental datasets used in the present study and fitted numerical results using the Kelvin-Voigt model are available online as supplementary information files with this paper.
